# Histopathological Findings in Adult Patients With Dyspepsia and Their Association With Helicobacter pylori Infection

**DOI:** 10.7759/cureus.50981

**Published:** 2023-12-23

**Authors:** Yossef H Ahmed, Rehab A Mohammed, Ibrahim K Alghamdi, Majdah F Alqahtani, Shaden N Alhelali, Intessar Sultan, Mayar I Badawy, Mahmoud M Barakat, Hanaa E Abozeid, Hanan L Mohammed

**Affiliations:** 1 Department of Clinical Research, Syreon Middle East, Dubai, ARE; 2 Department of Internal Medicine, Ibn Sina National College for Medical Studies, Jeddah, SAU; 3 Department of Internal Medicine, Faculty of Medicine for Girls, Al-Azhar University, Cairo, EGY; 4 Department of Medicine, Ibn Sina National College for Medical Studies, Jeddah, SAU; 5 Department of Clinical Pharmacy, Faculty of Pharmacy, Tanta University, Tanta, EGY; 6 Department of Medicine, Al-Azhar University, Cairo, EGY; 7 Department of Pathology, Ibn Sina National College for Medical Studies, Jeddah, SAU

**Keywords:** intestinal metaplasia, premalignant lesions, gastric biopsy, upper endoscopy, histopathology, helicobacter pylori

## Abstract

Background: *Helicobacter pylori* infection is prevalent among Saudi adults and has been linked to gastric cancer and other tumor-like conditions. We aimed to explore the pathological characteristics of endoscopic gastric biopsies among symptomatic adult Saudi patients and their relation to *H. pylori* infection.

Results: Among 151 gastric biopsies, gastritis was detected in 97 (64.2%) cases, chronic active gastritis in 26 patients (17.2%), duodenitis in 20 (13.2%) patients, and total metaplasia in 14 (9.3%) patients. *H. pylori* was detected in 83 cases (55%), with a recurrence or reinfection rate of 9.8%. The patients with *H. pylori* infection were considerably young (median age: 34 (IQR: 15) vs. 35.5 (IQR: 11), p = 0.024) and had a low frequency of epigastric pain (78.3% vs. 91.2%, p = 0.031), reflux/regurgitation (7.2% vs. 20.6%, p = 0.016), and dysphagia (4.85% vs. 14.7%, p = 0.037). However, they exhibited a higher incidence of chronic active gastritis (96.2% vs. 3.8%, p < 0.001) and intestinal metaplasia (85.7% vs. 14.3%, p = 0.015). Young age (OR = 1.09, 95% CI = 1.02-1.16, p = 0.011) and *H. pylori* infection (OR = 30.85, 95% CI = 3.26-291.60, p = 0.003) were identified as a positive predictor of intestinal metaplasia while heartburn (OR = 0.08, 95% CI = 0.01-0.58, p = 0.012) was a negative predictor.

Conclusion: *H. pylori* infection is prevalent among Saudi adults experiencing upper gastrointestinal symptoms and is associated with intestinal metaplasia. Infection rate and intestinal metaplasia were higher in patients with milder symptoms. Therefore, screening for *H. pylori* is highly recommended for Saudi individuals with upper gastrointestinal symptoms. Old age and *H. pylori* infection were identified as positive predictors of intestinal metaplasia, emphasizing the importance of early detection and management of *H. pylori* infection in the Saudi population.

## Introduction

*Helicobacter pylori*, an opportunistic, gram-negative, flagellated bacillus, has the ability to infect the stomach of around half of the global population across the world [1]. However, there is a wide variation in the prevalence of *H. pylori* infection across the world, with reported higher frequency in developing countries. In Saudi Arabia, *H. pylori* infections are high, affecting almost 80% of adults [1].

It is classified as a type I carcinogen that is linked to 89% of all gastric cancers [2], as well as mucosa-associated lymphoid tissue (MALT) lymphoma [3]. Long-standing infection can lead to preneoplastic pathological changes, atrophy, and intestinal metaplasia in half of the infected patients [4]. The spectrum of gastric pathology resulting from *H. pylori* infection includes peptic ulcer disease, chronic active gastritis, MALT lymphoma, and gastric adenocarcinoma [5,6].

Long-standing *H. pylori* infection is the primary cause of chronic active gastritis, where the organism colonizes the gastric antrum along with mucosal lymphocytic infiltration [7]. *H. pylori*-induced peptic ulceration occurs in the presence of pre-existing chronic superficial gastritis and is associated with increased gastric acid secretion [8]. The progression of gastric mucosal changes due to *H. pylori* infection follows a sequential pattern, starting from non-atrophic gastritis and culminating in preneoplastic lesions like atrophic gastritis, intestinal metaplasia, and dysplasia [9].

The diagnostic approach of *H. pylori* encompasses both invasive and non-invasive tests. Non-invasive techniques, such as urea breath tests, serology, and stool antigen tests, have limitations in distinguishing old from current infection, especially in endemic areas. Invasive tests relying on endoscopic procedures include histopathology, rapid urease test, and polymerase chain reaction. These invasive tests exhibit sensitivity and specificity levels exceeding 90% [10]. Therefore, the aim of the present study was to identify the characteristics of endoscopic gastric biopsy among symptomatic Saudi patients with *H. pylori* infection and to identify any preneoplastic changes.

## Materials and methods

Study design

The cross-sectional study included adult Saudi patients with an upper endoscopy and gastric biopsy for upper gastrointestinal symptoms between December 2022 and June 2023, performed in the Endoscopy Department of Ibn Sina College Hospital, Jeddah, Saudi Arabia.

Data collection

Data regarding age, gender, and upper gastrointestinal tract symptoms, including nausea, vomiting, heartburn pain, belching, upper gastrointestinal bleeding, lower chest pain, weight loss, and dysphagia, were obtained from the patients.

Histopathological technique

Based on hospital policy, upper endoscopy was carried out on overnight fasting patients using the “Pentax” forward viewing esophagus and gastro-duodenum, and gastric biopsy was taken from the antrum using local anesthesia. Biopsies were fixed in formalin and embedded in paraffin. The 5-µm thin tissue sections subsequently obtained were deparaffinized in xylene (Sinopharm, Beijing, China) to remove the wax and serially rehydrated by passing through decreasing concentrations of alcohol baths (100%, 90%, 80%, 70%) (Sinopharm), followed by staining in hematoxylin for three to five minutes and then washed in running tap water. Dipping in 1% acid alcohol (1% HCl in 70% alcohol) for a few seconds was done to remove excess dye from the section, followed by tap water washing. Counterstaining of sections was done by using 1% eosin for 10 minutes, followed by washing in tap water for one to five minutes. Dehydration in increasing concentration of alcohols was done, followed by clearing in two xylene baths. Sections were then mounted in dibutylphthalate polystyrene xylene (DPX), covered, and observed by light microscopy.

Ethical consideration

The study was approved by the Ethics Committee of Ibn Sina National College for Medical Studies, Jeddah, Saudi Arabia (IRB-10-04082022). Data were kept confidential.

Statistical analysis

Categorical variables were presented as numbers (percentage), while continuous variable (age) was presented as median (interquartile ranges, IQR) as it was not normally distributed. Nonparametric and chi-square tests were used for comparison between different groups. All data were analyzed using IBM SPSS Statistics version 22 (IBM Corp., Armonk, NY). The level of significance of the study was set at 0.05.

## Results

The study included 151 patients, 56 females (37.1%) and 95 (62.9%) males. Their median age was 34 (IQR: 14) years, ranging from 18 to 73 years. Out of the 151 patients with suspected upper GI disease, *H. pylori* infection was diagnosed histologically in 83 patients (55%). Among positive cases, eight (9.8%) had a documented history of *H. pylori* treatment, which might represent recurrence or reinfection. The most frequent clinical presentation of infected patients was epigastric pain in 65 (78.3%), followed by nausea/vomiting in 43 (51.8%), heartburn in 37 (44.6%), bleeding in 26 (31.3%), and dyspepsia in 19 (22.9%) cases. The least frequent presentations included reflux/regurgitation in six (7.2%), dysphagia in four (4.8%), and weight loss in three (3.6%) cases.

**Figure 1:**
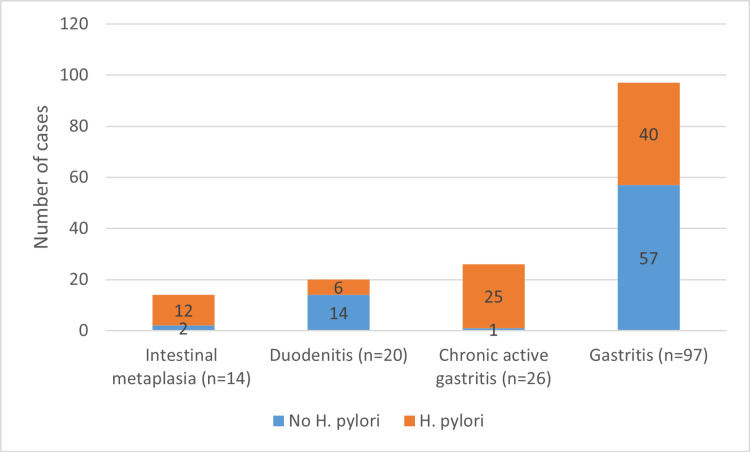
A stacked bar chart comparing those with and without histological diagnosis of H. pylori concerning their gastric biopsy results.

As seen in Table 1, infected patients tend to be younger (34 (IQR: 15) vs. 35.5 (IQR: 11) years, p = 0.024), they exhibited less symptoms of epigastric pain (78.3% vs. 91.2%, p = 0.031), reflux/regurgitation (7.2% vs. 20.6%, p = 0.016), and dysphagia (4.85 vs. 14.7%, p = 0.037), but higher frequency of gastritis (58.7% vs. 41.2%, p < 0.001) and intestinal metaplasia (85.7% vs. 14.3%, p = 0.015). The results of gastric biopsies are shown in Figure 1.

**Table 1:** Comparison of clinical data between patients with and without histological diagnosis of H. pylori infection.

		No, number = 68 (45%)			Yes, number = 83 (55%)		P
		Number	%	Number		%	
Gender	Females	24	35.3%	32		38.6%	0.680
	Males	44	64.7%	51		61.4%	
Age, median (IQR), years		35.5 (11)		34 (15)			0.024
Previous *H. pylori* infection	No	64	94.1%	74		90.2%	0.384
	Yes	4	5.9%	8		9.8%	
Epigastric pain	No	6	8.8%	18		21.7%	0.031
	Yes	62	91.2%	65		78.3%	
Heartburn	No	46	67.6%	46		55.4%	0.126
	Yes	22	32.4%	37		44.6%	
Nausea and or vomiting	No	36	52.9%	40		48.2%	0.561
	Yes	32	47.1%	43		51.8%	
Reflux or regurgitation	No	54	79.4%	77		92.8%	0.016
	Yes	14	20.6%	6		7.2%	
Dyspepsia	No	58	85.3%	64		77.1%	0.204
	Yes	10	14.7%	19		22.9%	
Dysphagia	No	58	85.3%	79		95.2%	0.037
	Yes	10	14.7%	4		4.8%	
Bleeding	No	56	82.4%	57		68.7%	0.054
	Yes	12	17.6%	26		31.3%	
Weight loss	No	64	94.1%	80		96.4%	0.510
	Yes	4	5.9%	3		3.6%	

**Figure 2:**
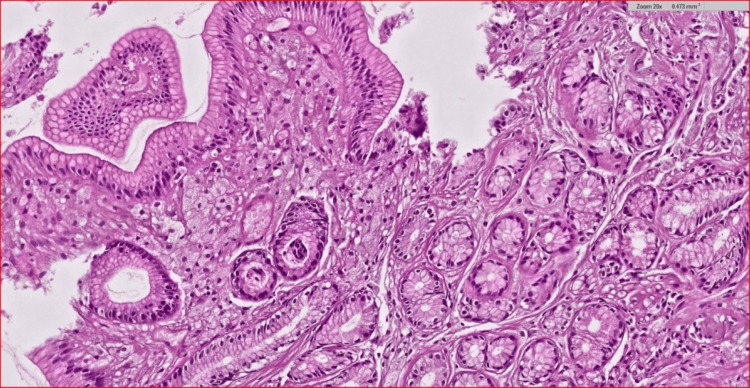
A case of chronic active gastritis in which gastric tissue is stained with hematoxylin & eosin at 100 magnification shows that the inflammatory infiltrate includes variable numbers of neutrophils within the lamina propria, with large numbers of plasma cells with increased numbers of lymphocytes and macrophages. The gastric mucosa shows variable degrees of intestinal metaplasia of many gastric glandular cells, a premalignant condition.

**Figure 3:**
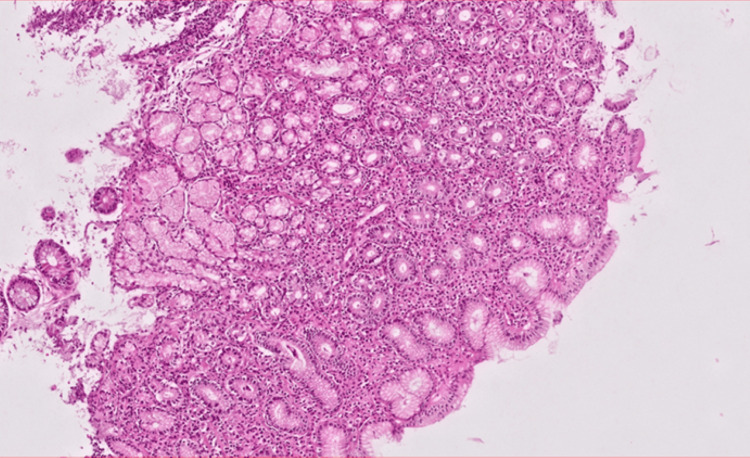
Gastric biopsy stained with hematoxylin & eosin at 400 magnification shows chronic H. pylori gastritis. The gastric mucosa shows an area of mucosal superficial ulceration with inflammatory infiltration formed of variable numbers of neutrophils, plasma cells, lymphocytes, and macrophages. Intraepithelial neutrophils and subepithelial plasma cells are characteristic of H. pylori gastritis. Mucous glands show areas of internalization as well as atrophic changes.

Among the total cases, 97 patients (64.2%) were diagnosed with gastritis, with *H. pylori* infection accounting for 57 cases (58.7%). Additionally, 20 cases (13.2%) were diagnosed with duodenitis, with only six (30%) having an *H. pylori* infection. Chronic active gastritis was detected in 26 patients (17.2%) (Figure 2), and 25 (96.2%) of them had *H. pylori* infection. The only pre-neoplastic lesion found was intestinal metaplasia, which was detected in 14 (9.3%) cases (Figure 3); *H. pylori* infection accounted for 12 cases (85.7%) of them. Compared to non-infected cases, patients with *H. pylori* infection had a significantly higher frequency of chronic active gastritis (96.2% vs. 3.8%, p < 0.001) and intestinal metaplasia (85.7% vs. 14.3%, p = 0.015).

Benign lesions found in the endoscopic gastric biopsies were one case of gastric xanthelasma among infected patients and two cases of duodenal Brunner's glands hyperplasia among non-infected patients. Only one case of bleeding peptic ulcer was diagnosed by the endoscope (biopsy cannot be taken because of bleeding).

Further analysis indicated that age (OR = 1.09, 95% CI = 1.02-1.16, p = 0.011) and *H. pylori* infection (OR = 30.85, 95% CI = 3.26-291.60, p = 0.003) were a positive predictor of intestinal metaplasia, while heartburn (OR = 0.08, 95% CI = 0.01-0.58, p = 0.012) was a negative predictor (Table 2).

**Table 2:** Predictors of gastric intestinal metaplasia among symptomatic patients.

	Odds ratio	95% confidence interval		P
		Lower	Upper	
Age	1.09	1.02	1.16	0.011
Gender	0.26	0.06	1.15	0.076
Epigastric pain	0.77	0.16	3.76	0.747
Heartburn	0.08	0.01	0.58	0.012
Nausea vomiting	0.27	0.06	1.27	0.096
Dyspepsia	0.34	0.04	3.14	0.340
Bleeding	0.11	0.01	1.12	0.062
*H. pylori* infection	30.85	3.26	291.60	0.003
Active chronic gastritis	0.43	0.08	2.19	0.308

## Discussion

Our results found that more than half of adult Saudi patients experiencing upper gastrointestinal symptoms were infected by *H. pylori* infection. It is important to note that our *H. pylori* detection rate involves symptomatic patients who underwent invasive diagnostic tests and, therefore, cannot be compared to the expected rate of 80% among the general Saudi population. However, an Indian study reported a rate of 62.0% [11]. Among the infected cases, 10% had a previous history of *H. pylori* eradication, representing either a recurrence of the original strain or reinfection by a new strain [12] and ultimately pointing to the possible emergence of drug resistance among Saudi patients.

In line with another study [13], our results demonstrated that chronic active gastritis is the most prevalent histologic pattern of *H. pylori* gastritis. None of the patients included had atrophic gastritis, MALT, or frank gastric cancer. In contrast to findings from other researchers who reported *H. pylori*-related peptic ulcers and dysplasia/carcinoma [14,15], we detected 14 cases with gastric intestinal metaplasia, which is the only detected pre-neoplastic gastric lesion in our patients. According to our results, this intestinal metaplasia is triggered by chronic *H. pylori* infection and age. In contrast to the previous research, the prevalence of *H. pylori* infection accounted for 38.6% of gastric intestinal metaplasia in Turkish patients [16]. Increasing age may be attributed to intestinal metaplasia because of prolonged gastric inflammation.

Regarding symptoms linked to infection, the most common were epigastric pain, nausea, and vomiting. The symptomatology of *H. pylori* infection varies widely, ranging from asymptomatic in most cases to mild self-limited symptoms or severe abdominal pain.

In the present study, gender did not appear to be a significant risk factor for infection or development of intestinal metaplasia. These findings are in concordance with others [11,17,18], but they differ from the results reported by Kaore et al. [19], who showed a higher prevalence in male gender. On the other hand, infection shows a significant trend toward the younger age group; this can be explained by the relatively young age of the cases incorporated in our study, with a median age of 34 years. This finding is in accordance with other studies [11,18,19] that reported more frequent infection in patients aged 20-40 years compared to the older age group. However, increasing age above 40 represented a positive factor for predicting preneoplastic intestinal metaplasia.

The present study has some limitations, including the study design that limits the evidence of a causal effect relationship between *H. pylori* infection and the development of gastrointestinal pathology.

## Conclusions

Our study highlights a significant prevalence of *H. pylori* infection among symptomatic adult Saudi patients. Though the prevalence of *H. pylori* chronic active gastritis and related abdominal symptoms is significant, no major gastrointestinal complications were detected apart from a substantial number of intestinal metaplasia. This preneoplastic lesion was linked to age and *H. pylori* infection. Based on our findings, we recommend screening for *H. pylori* infection and initiation of early treatment for primary prevention of gastric cancer in the young Saudi population aged 20 and 30 years before the development of preneoplastic conditions. Moreover, regular endoscopic follow-up should be provided to patients with preneoplastic lesions for secondary prevention.
